# Real-Time Estimation of Population Exposure to PM_2.5_ Using Mobile- and Station-Based Big Data

**DOI:** 10.3390/ijerph15040573

**Published:** 2018-03-23

**Authors:** Bin Chen, Yimeng Song, Tingting Jiang, Ziyue Chen, Bo Huang, Bing Xu

**Affiliations:** 1 Ministry of Education Key Laboratory for Earth System Modelling, Department of Earth System Science, Tsinghua University, Beijing 100084, China; bin.chen792@gmail.com (B.C.); ecnu_jtt@163.com (T.J.); 2 Department of Land, Air and Water Resources, University of California, Davis, CA 95616, USA; 3 Department of Geography and Resource Management, The Chinese University of Hong Kong, Shatin, Hong Kong, China; yimengsong@link.cuhk.edu.hk; 4 State Key Laboratory of Remote Sensing Science, College of Global Change and Earth System Science, Beijing Normal University, Beijing 100875, China; zychen@bnu.edu.cn; 5 Department of Geography, University of Utah, 260 S. Central Campus Dr., Salt Lake City, UT 84112, USA

**Keywords:** air pollution exposure, human mobility, mobile phone data, dynamic assessment

## Abstract

Extremely high fine particulate matter (PM_2.5_) concentration has been a topic of special concern in recent years because of its important and sensitive relation with health risks. However, many previous PM_2.5_ exposure assessments have practical limitations, due to the assumption that population distribution or air pollution levels are spatially stationary and temporally constant and people move within regions of generally the same air quality throughout a day or other time periods. To deal with this challenge, we propose a novel method to achieve the real-time estimation of population exposure to PM_2.5_ in China by integrating mobile-phone locating-request (MPL) big data and station-based PM_2.5_ observations. Nationwide experiments show that the proposed method can yield the estimation of population exposure to PM_2.5_ concentrations and cumulative inhaled PM_2.5_ masses with a 3-h updating frequency. Compared with the census-based method, it introduced the dynamics of population distribution into the exposure estimation, thereby providing an improved way to better assess the population exposure to PM_2.5_ at different temporal scales. Additionally, the proposed method and dataset can be easily extended to estimate other ambient pollutant exposures such as PM_10_, O_3_, SO_2_, and NO_2_, and may hold potential utilities in supporting the environmental exposure assessment and related policy-driven environmental actions.

## 1. Introduction

Air pollutants, especially fine particulate matters such as PM_2.5_ (particles with an aerodynamic diameter less than 2.5 μm), have been the focus of increasing public concern because of its strong relation with health risks [1,2]. Numerous epidemiologic studies have established robust associations between long-term exposure to PM_2.5_ and premature mortality associated with various health conditions—such as heart disease, cardiovascular and respiratory diseases, and lung cancer—that substantially reduce life expectancy [2,3,4,5,6,7]. With the unprecedented economic development and urbanization over the past three decades, the severe and widespread PM_2.5_ pollution has been one of the biggest health threats in China [8,9]. The Ministry of Environmental Protection reported that only eight of the 74 monitored cities meet China’s ambient air quality standards (annual mean: 35 μg/m^3^; and 24-h mean: 75 μg/m^3^) in 2014 [10], and the number of cities was only three in 2013 [11]. The country environmental analysis report from the Asian Development Bank shows that only <1% of 500 largest cities in China could meet the air quality guidance [12] (annual mean: 10 μg/m^3^; and 24-h mean: 25 μg/m^3^) suggested by the World Health Organization (WHO) [13].

Numerous studies have attempted to estimate ground PM_2.5_ concentration levels over the past decade. As ground monitoring stations provide temporally continuous records of air pollutant concentrations, the most straightforward method applied in previous researches is using the station-based PM_2.5_ observations directly to interpolate point- or surface-based PM_2.5_ concentration levels [14,15], thereby offering the near real-time estimations of PM_2.5_ pollution levels from local to regional scales. However, these stations are always limited in number and unevenly distributed, resulting in potential biases from interpolating local point-based measurements to surface-based estimations at a large spatial scale. Fortunately, the satellite-derived atmospheric aerosol optical depth (AOD) [16,17,18] has greatly advanced our understanding of spatially- and temporally- explicit changes of PM_2.5_ concentrations at both regional and global scales. Over the past decade, a number of pioneering works have been devoted to quantifying the relationship between satellite-based AOD retrievals and ground-measured PM_2.5_ concentrations. Here we categorize them into three major groups, (i) the chemical transport models. This type of models is based on characteristics of the vertical distribution and dispersal of aerosols, and it can further integrate aerosols’ components and the effects of other pollutants to predict ground-level PM_2.5_ concentrations. For example, Liu et al. [19] coupled the global atmospheric chemistry model (GEOS-CHEM) with AOD retrieved by the Multiangle Imaging Spectroradiometer (MISR) to map annual mean ground-level PM_2.5_ concentrations over the contiguous United States. By simulating factors that affect the relation between AOD and PM_2.5_, van Donkelaar et al. [17] estimated a global field of surface PM_2.5_ concentrations with the AOD retrieved from both the Moderate-resolution Imaging Spectroradiometer (MODIS) and MISR observations. (ii) The semi-empirical models. This type of models is generally based on the modeling of the AOD-PM_2.5_ relationships by incorporating environmental factors. For example, several semi-empirical models have been developed ranging from simple linear relationships to complex nonlinear relationships involving meteorological and geographic variables [20,21]. (iii) The statistical regression models. This type of models is based on statistical regressions by regarding ground-based PM_2.5_ measurements as the dependent variables, and the satellite-based AOD retrievals and other factors including topography, land cover/use types, humidity, temperature, wind speed, wind direction, vertical visibility, and the height of boundary layer, etc., as the independent variables [22,23,24,25,26,27]. Despite the integration of satellite- and station-based observations has proven to be useful in improving the retrieval accuracy of PM_2.5_ concentrations, the available datasets are still with a coarse temporal resolution from daily, to monthly or yearly scales, rather than depicting the spatiotemporal variation of PM_2.5_ concentrations within a day.

Another critical issue relating to the estimation of population exposure to PM_2.5_ pollutants is that most of existing exposure assessments always regard population as static, without considering the temporal dynamics of population distribution [14,28]. Currently, demographic data based on administrative units are the most widely used data source for estimating air pollution exposure risks. It provides accurate population census information over a certain time period based on the smallest administrative unit (i.e., census block) and often includes kinds of socio-economic attributes such as age, gender, education, and income. However, such kind of data has limitations for estimating the real-time exposure risks to air pollutants since it just regards population as a homogeneous entity for each census block without diving into the spatial heterogeneity of population distribution. More importantly, it does not consider spatiotemporal dynamics of the human mobility due to the very low updating frequency. In contrast, recent studies have demonstrated the necessity of considering spatiotemporal variability of air pollution and human mobility in exposure assessments [14,29,30,31]. That is because, first, air pollution concentrations are not only spatially varied but also changing across temporal scales from minutes to hours, and second, population exposure to air pollutants is actually determined by both the specific location and how much time spent on that location, rather than the assumption that people move within regions of generally the same air quality throughout a day or other time periods. Thus, how to obtain real-time estimations of population exposure to PM_2.5_ concentrations is urgently needed for instant or short-time assessments (e.g., hourly or short-term PM_2.5_ concentrations are more relevant to vulnerable population groups than the daily or monthly concentrations on average [14]) and cumulative exposure effects (the aggregation of short-term assessments is more robust than the monthly or annual average).

Addressing these ubiquitous challenges, more information on human space-time location is required. Some of previous studies have tried to use surveying data, such as travel questionnaire surveys, personal GPS or smart sensor based devices [14,31,32] to delineate how an individual move in the city during his/her daily life. For example, Lu and Fang [32] used the GPS-equipped portable air sensor to measure air pollutant intakes in individual’s immediate surroundings and space-time movement trajectories in Huston, Texas. However, their high expenses and limited samples within local areas barricade the data availability. The alternative approaches are to use mathematical models to simulate population mobility patterns, such as gravity model [33] and radiation model [34]. This kind of methods allow us to draw more quantitative conclusions from a larger population size, but their results are only valid for situations with similar initial parameters in the simulation process [29]. Recently, Park and Kwan [14] simulated 80 possible daily movement trajectories based on daily trip distribution data from the Congestion Management Program Report to reflect the actual commuting tendency of Los Angeles (USA) county residents, and estimated exposure risks by considering the interactions between air pollution and individuals’ location. However, such kind of studies are still constrained to limited spatial and temporal scales. With the rapid growth of mobile internet, especially the location-based services of applications (apps) in the smartphones, it makes us possible to access direct spatiotemporal records of human activities [35,36]. Additionally, the high correlation between the mobile-phone locating-request records and the spatiotemporal characteristics of human activities has been revealed by many studies [37,38,39]. A growing number of studies have started to use mobile phone data in the field of environmental exposure assessments [29,30,40]. For example, Dewulf et al. [29] collected mobile phone data of approximately five million mobile users in Belgium to calculate the daily exposure to NO_2_. Gariazzo et al. [30] conducted a dynamic city-wide air pollution (NO_2_, O_3_, and PM_2.5_) exposure assessment by using time resolved population distributions derived from mobile phone traffic data, and modelled air pollutants concentrations. Yu et al. [40] combined cell phone location data from 9886 SIMcard IDs in Shenzhen, China to assess the misclassification errors in air pollution exposure estimation. Although all these pioneering studies highlight the promising advantages of incorporating population dynamics in estimating air pollution exposure, the available datasets are still limited to sample sizes and spatiotemporal scales due to the cost and time for collecting fine-resolution data, data privacy and confidentiality issues, and computational complexities [41].

To investigate the nationwide PM_2.5_ concentration risks for population in China, spatially explicit and temporally continuous studies are needed to detect hotspots, estimate vulnerability, and assess population exposure at finer temporal scales. In this paper, we propose a novel approach to achieve the real-time estimation of population exposure to PM_2.5_ by integrating mobile-phone locating-request (MPL) big data and station-based PM_2.5_ observations. Compared with previous studies regarding ambient pollution exposure assessments, it has the following highlights. First, the proposed method introduces the dynamics of population distribution into the nationwide exposure estimation, thereby providing an improved way to better assess the actual exposure risk to PM_2.5_ at different temporal scales. Second, to the best of our knowledge, it is the first time to provide the real-time estimation of nationwide population exposure to PM_2.5_ at pixel-based level (~1.2 km) in China. Third, the proposed method and dataset can be easily extended to estimate other ambient pollutant exposures such as PM_10_, O_3_, SO_2_, and NO_2_, and may hold potential utilities in supporting the environmental exposure assessments and related policy-driven environmental actions.

## 2. Materials and Methods

### 2.1. Ground-Station PM_2.5_ Measurements

**Figure 1:**
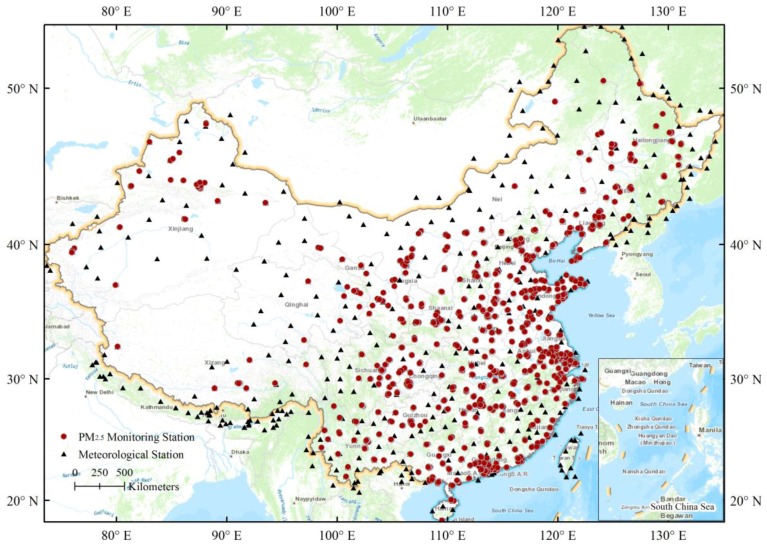
Spatial distribution of nationwide monitoring stations for PM_2.5_ concentrations (red dots) and meteorological stations (black triangles) in China.

Hourly ground-station PM_2.5_ measurements from 1 March to 31 March 2016 were collected from the official website of the China Environmental Monitoring Center (http://113.108.142.147:20035/emcpublish/). According to the Chinese National Ambient Air Quality Standard (CNAAQS), the station-based PM_2.5_ data in China were obtained using the tapered element oscillating microbalance method (TEOM) or the beta-attenuation method, combined with the periodic calibration. In this study, we used a total of 1465 monitoring stations (Figure 1) that have been established in all provinces for monitoring ambient air quality.

### 2.2. Ground-Station Meteorological Measurements

Ground-station meteorological variables, including air temperature (AT), surface wind speed (WS), and horizontal visibility (VIS) were used from Global Telecommunication System (GTS) established by World Meteorological Organization (https://rda.ucar.edu/datasets/ds461.0/). In this study, the 3-h measurements (from 2:00 a.m. to 23:00 p.m. local time) from 411 stations in China and 128 stations within the 0.01-degree buffer zones around the boundary of China (Figure 1) were collected from 1 March to 31 March 2016.

### 2.3. Mobile Phone Locating-Request Big Data

By retrieving real-time locating requests from mobile phone users’ activities in apps, the mobile phone locating-request (MPL) data was used in this study to monitor human movement. The MPL data are from Tencent big data platform in China, which is one of the largest Internet service providers both nationwide and worldwide. All of the MPL data are produced by active smartphone users using apps, which have been enabled to report real-time locations from the mobile devices. Due to the widespread usage of Tencent apps (e.g., WeChat, QQ, Tencent Map, etc.) and their location-based services, the daily locating records have reached 36 billion from more than 450 million users globally in 2016 [42]. Thus, the MPL big data can be represented as an indicator to characterize human activities and population distribution in a fine spatiotemporal scale. The Tencent MPL dataset used in this study was collected from 1 March to 31 March 2016 via the application program interface (API) from the Tencent big data platform (http://heat.qq.com). The original Tencent MPL dataset was recorded by aggregating the real-time locations of active apps users every five minutes within a mesh grid at a spatial resolution of 30 arc-second (~1.2 km). All the information regarding users’ identities and privacies were removed in this publicly available dataset.

### 2.4. Population Census Data

The latest city-level population census of China in 2014 obtained from the national scientific data sharing platform for population and health (http://www.ncmi.cn/) was used in this study. This dataset was established and maintained by infectious disease network reporting system, and it was derived based on population census released by the State Statistics Bureau. It collected all population census including permanent resident and registered resident at the county level by gender and age group since 2004.

### 2.5. Estimation of Spatiotemporal Continuous PM_2.5_ Concentrations

Due to the difference in geographic locations between PM_2.5_ monitoring stations and meteorological stations, all datasets were processed to be consistent in spatial and temporal domains. The meteorological variables were first interpolated by ordinary Kriging method [43] to obtain data that covering the entire study area with a spatial resolution of 30 arc-second (~1.2 km). To mitigate the interpolation biases, we averaged all meteorological observations with a 30 arc-second search radius around each PM_2.5_ monitoring station, and then assigned the result to the corresponding PM_2.5_ monitoring station. In addition, the widely used Geographically Weighted Regression (GWR) model [44] with adaptive Gaussian bandwidth was adopted to build the statistical relationship between meteorological variables and PM_2.5_ concentrations. Specifically, we grouped all variables within a month into 8 time points (i.e., from 2:00 a.m., 5:00 a.m., …, 23:00 p.m.), and then developed 8 GWR models for each time point in this study as follows:

(1) $$PM_{2.5,i,t}=\beta_{0,i,t}+\beta_{1,i,t}VIS_{i,t}+\beta_{2,i,t}AT_{i,t}+\beta_{3,i,t}WS_{i,t}$$

where *PM*_2.5,*i,t*_ denotes the PM_2.5_ concentration at the location *i* at time *t*, *VIS_i,t_*, *AT_i,t_*, and *WS_i,t_* denote the visibility (m), air temperature (°C), and surface wind speed (m/s), respectively, at location *i* at time *t*. *β*_0,*i,t*_, *β*_1,*i,t*_, *β*_2,*i,t*_, and *β*_3,*i,t*_ are corresponding regression coefficients at location *i* at time *t*.

A 10-fold validation analysis [45] was adopted to evaluate the modeling performance by comparing the estimated and measured PM_2.5_ concentrations (details can be found in Supplementary Materials). With the iterative cross validations, the optimal coefficients in each time point were retrieved to interpolate the entire study areas with a spatial resolution of 30 arc-second (~1.2 km), and then were used to estimate gridded PM_2.5_ concentrations.

### 2.6. Estimation of Real-Time Population Distribution by Integrating MPL and Census Data

The mobile phone locating-request (MPL) data can be served as an indicator to delineate the spatiotemporal pattern of population distribution, however, the MPL data do not represent the actual population sizes. In this study, we first aggregated the 5-min MPL data into 3-h MPL data, making its temporal resolution consistent with that of the estimated PM_2.5_ concentrations, and calculated the pixel-based population density using the MPL data, and then applied the MPL-based population density map to downscale the census data. Consequently, we can obtain the 3-h pixel-based population approximations. Given the difference of physical environment and socio-economic development in various areas of China, downscaling the MPL data with population census at the national scale will undoubtedly result in the underestimation of population in under- and less-developed areas and overestimation of population in those developed areas. To solve this problem, we decided to estimate real-time population distribution by integrating MPL and census data at the city level. The 3-h MPL map was used to redistribute the census data for each city by Equations (2) and (3), under the assumption that the inter-city mobility will not dramatically influence the total population of a city within a short time window. Finally, we could obtain the 3-h pixel-based population approximation for each city, and then conducted the image mosaic to produce the 3-h national-scale population distribution map in China.

(2) $$W_{ij}=\frac{p_{ij}}{\sum_{i=1}^{n}p_{ij}}$$

(3) $$pop_{ij}=TR\times W_{ij}$$

where *p_i,j_* is the amount of locating-request times within the *i*-th pixel at the hour *j*, *n* is the total number of pixels within a city, *W_i,j_* is the weight for redistributing population and *TR* is the total population in the city from the census data. *Pop_i,j_* denotes the population approximation in the *i*-th pixel at the hour *j*.

### 2.7. Real-Time Estimation of Population Exposure to PM_2.5_

Since the levels of PM_2.5_ concentration and population distribution are spatially and temporally varied, here we adopted the population-weighted metric (Equation (4)) to estimate the real-time exposure risks to PM_2.5_ concentrations, which was likely to be more representative of population exposure to PM_2.5_ across different temporal scales [46]:

(4) $$PWP=\sum_{i=1}^{N}\left(pop_{i}\cdot pm_{i}\right)/\sum_{i=1}^{N}pop_{i}$$

where *pop_i_* and *pm_i_* denote the population and PM_2.5_ concentration level in the *i*-th pixel, *N* is the total number of pixels within the corresponding administrative unit. *PWP* is the population-weighted PM_2.5_ concentration level for the targeted administrative unit.

With the PM_2.5_ concentrations and population distribution estimated in previous sections, we could integrate them based on Equation (4) to provide the estimation of population exposure to PM_2.5_ with a 3-h updating frequency, thereby being able to track the real-time dynamics of exposure risks by considering the spatiotemporal variation of PM_2.5_ concentration and population distribution.

### 2.8. Estimation of Cumulative Inhaled PM_2.5_

PM_2.5_ concentration causes acute and chronic adverse effects on human health mainly by means of inhalation exposure. To our understanding, deriving the estimations of cumulative inhaled PM_2.5_ masses will be one of the most important prerequisites to model the accurate relationship between PM_2.5_ exposure and human health [47,48,49]. Thus, we proposed to incorporate human respiratory volume and the spatiotemporal variation of PM_2.5_ concentration and population density to present a better estimation of cumulative inhaled PM_2.5_:

(5) $$InPM_{2.5}=\sum_{i=1}^{N}\sum_{t=1}^{T}p_{i}(t)\cdot h_{i}\cdot d_{i}(t)\cdot m(t)+p_{i}(t)\cdot h_{i}\cdot \left(1-d_{i}(t)\right)\cdot m(t)\cdot \alpha$$

where *p_i_* and *h_i_* denote the population and the inhaled volume of air for the *i*-th age group, *N* is the total number of the age group. *t* denotes the time (hours in this study), *m*(*t*) denotes the PM_2.5_ concentration level at time *t*, *T* is the target temporal period, *d_i_* is the percentage of outdoor population, α is the outdoor-indoor ratio of PM_2.5_ concentration.

However, recent advances regarding the outdoor-indoor ratio of PM_2.5_ concentrations are all limited to local scales for the purpose of experimental tests [50], as it is difficult to acquire such valid observations relating this ratio on a large scale. More importantly, the outdoor-indoor ratio is influenced by several factors such as geographic location, building structures, and living habits. In addition, the inhaled volume of air is also different, not only in terms of age differences but of physical activities, gender, and size, all of these factors would affect the inhaled value [51,52]. Thus, we have to simplify the ideal model in Equation (5) for being suitable to nationwide estimates of cumulative inhaled PM_2.5_ masses by neglecting the difference between outdoor and indoor PM_2.5_ concentration exposure and the inhaled volume of air among different age groups, gender, and other related factors. In this way, we can directly obtain the estimation of cumulative inhaled PM_2.5_ masses using the following equation:

(6) $$InPM_{2.5}^{'}=\sum_{t=1}^{T}p_{i}(t)\cdot h\cdot m(t)$$

where $InPM_{2.5}^{'}$ denotes the cumulative inhaled PM_2.5_ mass from the simplified model, and *h* denotes the empirical inhaled volume of air. A measurement conducted by Adams [51] based on 200 individuals showed that the hourly average volume of air breathed by adults when they are sitting or resting were ranging from 0.42 to 0.63 m^3^ (i.e., 10.08 to 15.12 m^3^/day), and the volumes for walking were from 1.20 to 1.44 m^3^/h, and for running were from 3.10 to 3.48 m^3^/h. Thus, the average inhaled volume of air for an individual is assumed to be 15 m^3^/day in this study [52].

### 2.9. Comparison of Exposure Assessments from the MPL-Based and Census-Based Methods

In order to investigate whether the improvement of incorporating dynamic population distributions does make a difference in the exposure assessment, we intuitively compared the MPL-based and census-based calculations of cumulative inhaled PM_2.5_ masses and population-weighted PM_2.5_ exposure concentrations in China’s 359 cities across different temporal scales (i.e., 3-h, 1-day, 1-week, and 1-month). For each city, the population from the census data was directly used in the census-based method, while the redistributed population dynamics was used in the MPL-based method.

## 3. Results

### 3.1. Different Facets of Population Exposure to PM_2.5_

**Figure 2:**
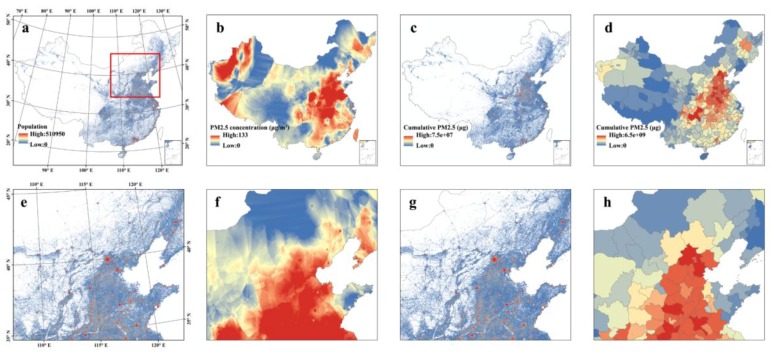
Different facets of population exposure to PM_2.5_. (**a**) Map of population distribution in China on 1 March 2016 (11:00 a.m.). (**b**) Map of PM_2.5_ concentration levels in China on 1 March 2016 (11:00 a.m.). (**c**) Map of cumulative inhaled PM_2.5_ masses in China based on the MPL data on 1 March 2016. (**d**) Map of cumulative inhaled PM_2.5_ in China based on the census data on 1 March 2016. (**e**–**h**) show the insets from (**a**–**d**) for part of the Northern China.

The spatiotemporal integration of PM_2.5_ concentration and population density was used to produce thematic information that document different facets of population exposure to PM_2.5_. Figure 2 shows an extracted example from the time-series analysis of population exposure to PM_2.5_ in China.

Figure 2a shows the real-time nationwide estimation of population distribution (11:00 a.m.) on 1 March 2016, which is derived by integrating MPL and census data at a city-level scale in Section 2.6. The intensity represents the specific population number in each gridded pixel with stretched colors from blue to red denoting varied population size. Figure 2b shows the real-time nationwide estimation of PM_2.5_ concentrations (11:00 a.m.), which is derived from incorporating ground-station PM_2.5_ measurements and meteorological variables based on GWR models in Section 2.5. Figure 2c shows the nationwide estimation of 24-h cumulative inhaled PM_2.5_ masses. Figure 2d shows the estimation of 24-h cumulative inhaled PM_2.5_ masses based on the census data. Figure 2e–h show insets from Figure 2a–d for part of the Northern China as a zoomed visualization in different facets of population exposure to PM_2.5_ concentrations.

### 3.2. Temporal Dynamics of Population Exposure to PM_2.5_

**Figure 3:**
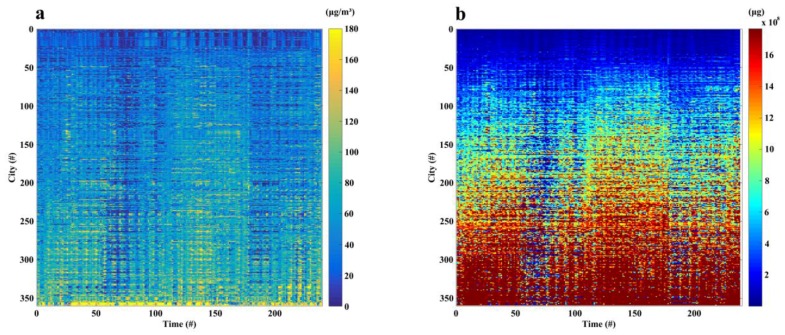
The estimated population-weighted PM_2.5_ concentrations (**a**) and cumulative inhaled PM_2.5_ masses (**b**) for 359 cities in China with every 3 h from 1 March to 31 March 2016. Note that the *x* axis represents the time from the first 3-h (2:00 a.m. 1 March 2016) to the last 3-h (23:00 p.m. 31 March 2016), and *y* axis represents the order of 359 cities.

In the form of Figure 2a–c, we can also provide the temporal variation of population, PM_2.5_ concentrations, and cumulative inhaled PM_2.5_ masses with a 3-h temporal resolution from 1 March to 31 March 2016. In this way, the pixel-based dynamics of population exposure to PM_2.5_ concentrations at the nationwide scale with a nearly real-time updating frequency (i.e., 3-h in this study) were retrieved. In order to better present the experimental results with an entire month in March 2016, we further aggregated the pixel-based estimations into 359 cities in this study. Results demonstrate that both the population-weighted PM_2.5_ concentrations (Figure 3a) and cumulative inhaled PM_2.5_ masses (Figure 3b) exist distinguished diurnal and daily variations, which also verify the necessity of considering the spatiotemporal variability of both air pollution and population distribution in air pollution exposure assessments.

### 3.3. Comparison of Exposure Assessment Methods

**Figure 4:**
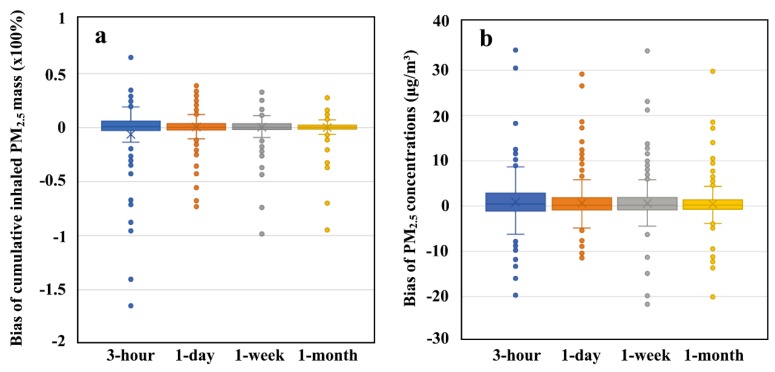
The biases of cumulative inhaled PM_2.5_ mass (**a**) and the per capita PM_2.5_ exposure concentration (**b**) between the MPL-based estimations and the census-based estimations in China’s cities across different temporal scales.

From the visual inspection from Figure 2c,d, it can be found out that the MPL-based method yields the gridded cumulative inhaled PM_2.5_ masses, whereas the census-based assessments are only based on administrative units (cities in this study), which informs us that the MPL-based method improves the spatial resolution of basic cells from administrative units to gridded pixels in exposure assessments. In addition, by comparing the cumulative inhaled PM_2.5_ masses and population-weighted PM_2.5_ exposure concentrations in China’s 359 cities across different temporal scales, results in Figure 4 show that without introducing the dynamics of population distribution into the exposure assessment, the maximum biases (over- or under- estimation) of cumulative inhaled PM_2.5_ mass reach to over 100% across different temporal scales. Meanwhile, the maximum biases of population-weighted PM_2.5_ concentrations will be approximately 30 μg/m^3^. By aggregating the experimental tests in China’s 359 cities from 1 March to 31 March 2016, the biased percentage between the MPL-based and the census-based estimations will be the level of 14.9% (3-h), 5.8% (1-day), 4.7% (1-week), and 3.9% (1-month) on average.

## 4. Discussion

Compared with previous methods for air pollution exposure assessment, the proposed method in this study considered well the spatiotemporal variability of both population distribution and PM_2.5_ concentration levels, thereby contributing to a better exposure assessment. The relative reasonability of our method may be due to the following strengths. First, the spatiotemporal variability of PM_2.5_ concentrations and population distribution are incorporated in air pollution exposure assessments. Given that the level of PM_2.5_ concentrations is continuously changing over space and time and human beings are also mobile across spatiotemporal scales [14], both of these dynamic characteristics and their interactions at finer spatiotemporal scales should be well considered to estimate population exposure risks. However, many previous studies always used the census data with the assumption that people are non-mobile or moving within regions of generally the same air quality throughout a day or other time periods, thus leading to considerable biases in actual air pollution exposure assessments. In reality, people in different areas experience different levels of PM_2.5_ concentrations across different temporal scales. In order to characterize the interaction between population dynamics and PM_2.5_ concentrations, here we used the mobile-phone locating-request (MPL) big data to quantify the dynamics of population distribution. By integrating the MPL and census data, we then derived real-time pixel-based population dynamics at the nationwide scale. Combing this nationwide population dynamic information and surface-based PM_2.5_ concentrations simultaneously will be of great importance to assess the actual population exposure to PM_2.5_ at different temporal scales. Second, the characterized dynamics of PM_2.5_ concentrations and population dynamics in the proposed method keep a consistent spatiotemporal scale. The MPL data used in this study were initially retrieved at a 5-min updating temporal resolution from the Tencent big data platform. We further aggregated the 5-min updating MPL data into 3-h synthetic data, making it temporally comparable to the updating frequency of the nationwide surface-based PM_2.5_ concentrations. Meanwhile, the spatial resolution of PM_2.5_ concentrations is also set to be with a 30 arc-second (~1.2 km) spatial resolution, which is the same with that of MPL data. These efforts contribute much to achieving near real-time (3-h) estimates of national population exposure to PM_2.5_ at the pixel-based level in China. Third, the presented model incorporated human respiratory volume and the spatiotemporal variation of PM_2.5_ concentration and population density to estimate cumulative inhaled PM_2.5_ masses. It will contribute to advancing the development of modelling the relationship between PM_2.5_ exposures, health risks, and life expectancies quantitatively.

Besides PM_2.5_, the ground monitoring stations are always coupled with sensors measuring other air pollutants such as PM_10_, SO_2_, NO_2_, and O_3_. With the similar framework by integrating mobile phone big data and air pollutant concentrations, the proposed method can also be customized to estimate population exposure risks to these ambient pollutants in China. Compared with the census-based method, the MPL-based method can yield near real-time estimations of population exposure to ambient pollutants. That is, we can achieve the estimation of air pollution exposure risks at any specific location and time on a large scale by combining the spatiotemporal variability of population distribution and air pollutant concentrations. By aggregating the short-term exposure assessments into longer temporal scales, we can also derive more robust and reliable estimations related to the chronic effects from air pollutants. Additionally, the proposed framework can be also applied to estimate the real-time number of people exposed to poor air quality as a result of updating the population distribution and air pollutant concentrations.

Meanwhile, some potential concerns regarding the implementation of the proposed method should be pointed out. First, in order to redistribute the census data to derive real-time population dynamics using the MPL data, we assume that the total population of each administrative unit (359 cities in this study) is constant since the inter-city mobility (the trade-off of inflow and outflow population) will not dramatically influence the total population of a city within a short time window. Thus, human movements and migrations across administrative units are neglected in this study. Second, volunteer-produced geospatial big data, such as MPL records in this study tend to leave out some population groups of the society because the children, the elderly, and the poor are less-frequent active users. Nevertheless, such data can still well quantify actual population distribution patterns [35,37,38] because of the massive volumes of data records. Here we take the MPL records in China on 1 March 2016 for example, the total number of locating-request records reaches 1.71 billion. By aggregating all MPL records from 1 March to 31 March 2016, the total number of locating-request records will be approximately 60 billion, thereby providing a robust measurement of population dynamics. Third, although the nationwide PM_2.5_ concentrations used in this study are estimated by incorporating the meteorological variables and ground-based PM_2.5_ measurements with the GWR models, the spatial interpolations are still the limits to affect the estimation accuracy in areas without sufficient inputs of station-based variables. As a result, even there is much greater spatial variations in the population data, there will be relatively less spatial variations in PM_2.5_ concentrations, which may lead to no significant impacts on the exposure assessments. However, with the comparison of exposure assessments between the MPL-based and the census-based methods, we can still figure out considerable differences. Thus, if we can further improve the estimation of PM_2.5_ concentrations, such as developing spatial-temporal integrated method by combing satellite-based and station-based observations guided with the diurnal change pattern of PM_2.5_ concentrations, land cover/use types, landscape topography, and related meteorological variables, the combination of the mobile phone big data and the improved air pollutant concentrations will contribute to a more reliable exposure assessment. Finally, the simplified model without considering outdoor-indoor ratio of PM_2.5_ concentrations and the difference of inhaled volumes of air among different population groups may be biased to the assessment of actual cumulative inhaled PM_2.5_ masses. As the Tencent-based MPL dataset was recorded by aggregating the real-time locations of active apps users within a mesh grid at a spatial resolution of 30 arc-second (~1.2 km) without differentiating individual’s moving trajectories and population groups, it was impractical to apply empirical parameters into the exposure assessment at a nationwide scale since the outdoor-indoor ratio of PM_2.5_ concentrations is influenced by several factors such as geographical locations, building materials, living habits, and so on. Similarly, the gridded MPL data without tracking individuals’ trajectories also prevented us from considering the commuting patterns or choices of different transports. However, the MPL dataset represents the unique data source having the best spatial resolution with real-time updating population distribution we can access right now. Meanwhile, the estimates in the experimental test also represent the trade-off between over- and under-estimated cumulative inhaled PM_2.5_ masses. On the one hand, these estimates are the highest estimates of cumulative inhaled PM_2.5_ masses since we do not consider the situations that people are with indoor environments or commuting transportations. On the other hand, the cumulative inhaled PM_2.5_ masses could be even higher because we use the constant value representing a low level of the inhaled air volume for an adult without considering factors such as physical activity, gender, and size [51]. Thus, these over and under estimates help balance each out in terms of cumulative inhaled PM_2.5_ masses to provide the general assessment at large scales.

## 5. Conclusions

This study sought to combine mobile phone big data and station-based PM_2.5_ measurements to achieve real-time estimations of population exposure to PM_2.5_ concentrations in China. The results showed that the proposed method can well quantify dynamics of the real-time population distribution and yield the estimation of population exposure to PM_2.5_ concentrations and cumulative inhaled PM_2.5_ masses with a 3-h updating frequency. This study provides a novel framework for environmental exposure assessments by considering the spatiotemporal variability of both population distribution and PM_2.5_ concentrations, which can also be customized to estimate other ambient pollutant exposure risks. These findings and methods may hold potential utilities in supporting the environmental exposure assessment and related policy-driven environmental actions.

## Supplementary Materials

The following are available online at http://www.mdpi.com/1660-4601/15/4/573/s1, Table S1. Accuracy of the fitting and 10-fold cross-validation for eight periods. Figure S1: Scatterplots of the observed and predicted PM_2.5_ for eight-time periods.
