# Supplementary material for: Real-Time Estimation of Population Exposure to PM2.5 Using Mobile- and Station-Based Big Data
Source: Int J Environ Res Public Health. 2018 Mar 23;15(4):573. doi: 10.3390/ijerph15040573 (PMC5923615; doi:10.3390/ijerph15040573)
Supplement: Supplementary file 1 [file ijerph-15-00573-s001.pdf]

Supplementary Information

Real-Time Estimation of Population Exposure to $PM_{2.5}$ Using Mobile- and Station-Based Big Data

Bin Chen [1,2], Yimeng Song [3], Tingting Jiang [1], Ziyue Chen [4], Bo Huang [3,*], and Bing Xu [1,4,5,*]

[1] Ministry of Education Key Laboratory for Earth System Modelling, Department of Earth System Science, Tsinghua University, Beijing 100084, China; bin.chen792@gmail.com (B.C.); ecnu_jtt@163.com (T.J.)

[2] Department of Land, Air and Water Resources, University of California, Davis, CA 95616, USA

[3] Department of Geography and Resource Management, The Chinese University of Hong Kong, Shatin, Hong Kong; yimengsong@link.cuhk.edu.hk (Y.S.)

[4] State Key Laboratory of Remote Sensing Science, College of Global Change and Earth System Science, Beijing Normal University, Beijing 100875, China; zychen@bnu.edu.cn (Z.C.)

[5] Department of Geography, University of Utah, 260 S. Central Campus Dr., Salt Lake City, UT 84112, USA

* Correspondence: bohuang@cuhk.edu.hk (B.H.); bingxu@tsinghua.edu.cn (B.X.); Tel.: +852-3943-6536 (B.H.); Tel.: +86-10-6279-3906 (B.X.)

The Performance Validation of GWR Models

In this study, we have 11675 samples in total for training and testing GWR models. That is, there will be around 1459 samples used for modelling during each period. For each model, about 1313 samples are used to develop GWR models and the rest are for a cross validation to test the model's performance. The fitting results of GWR models and the accuracy of 10-fold cross-validation are showed in Table S1. Specifically, for the fitting session among eight time periods, the average r-square (R^2) between the predicted and observed $PM_{2.5}$ concentrations is 0.56, the root-mean-square-error (RMSE) is around 20.87 μg/m³, and the mean-absolute-error (MAE) is 9.63 μg/m³. In contrast, for the validation session, the average R^2, RMSE, and MAE is 0.47, 22.65 μg/m³, and 10.63 μg/m³, respectively. Figure S1 also presents the 1-to-1 scatterplots for 10-fold cross-validations.

Table S1. Accuracy of the fitting and 10-fold cross-validation for eight periods.

	R^2		RMSE(μg/m³)		MAE(μg/m³)	
	**Fitting**	**Validation**	**Fitting**	**Validation**	**Fitting**	**Validation**
Hour 2	0.53	0.43	22.39	24.38	10.58	11.68
Hour 5	0.45	0.33	23.25	25.49	8.83	9.68
Hour 8	0.53	0.43	20.24	21.93	8.06	9.02
Hour 11	0.60	0.53	21.13	22.67	9.70	10.60
Hour 14	0.59	0.50	18.66	20.32	9.26	10.25
Hour 17	0.58	0.50	21.71	23.44	10.46	11.57
Hour 20	0.61	0.54	19.03	20.51	9.39	10.29
Hour 23	0.57	0.48	20.58	22.50	10.77	11.93
Average	0.56	0.47	20.87	22.65	9.63	10.63

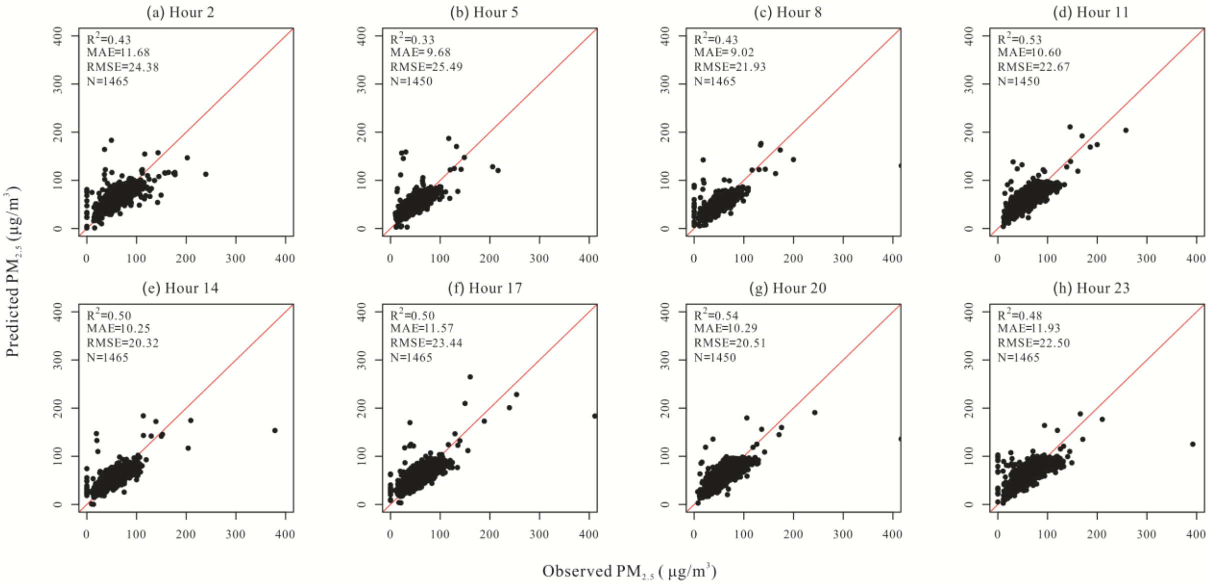

Figure S1. Scatterplots of the observed and predicted PM2.5 for eight time periods.
